# Head and neck cancer risk factors in the French West Indies

**DOI:** 10.1186/s12885-021-08787-4

**Published:** 2021-09-30

**Authors:** Aviane Auguste, Clarisse Joachim, Jacqueline Deloumeaux, Stanie Gaete, Léah Michineau, Cécile Herrmann-Storck, Suzy Duflo, Danièle Luce

**Affiliations:** 1 Univ Rennes, Inserm, EHESP, Irset (Institut de recherche en santé, environnement et travail) -UMR_S 1085, F-97100 Pointe-à-Pitre, France; 2 Martinique Cancer Registry, UF 1441 Registre des cancers, Pôle de Cancérologie Hématologie Urologie Pathologie, University Hospital of Martinique, Fort-de-France, Martinique France; 3 General Cancer Registry of Guadeloupe, University Hospital of Guadeloupe, Pointe-à-Pitre, Guadeloupe France; 4 Biological Resource Center Karubiotec™, BRIF n° KARUBIOTEC-G, UA-00971 Pointe-à-Pitre, Guadeloupe France; 5 Laboratory of Microbiology, University Hospital of Guadeloupe, Pointe-à-Pitre, Guadeloupe France; 6 Department of Oto-Rhino-Laryngology and Head and Neck Surgery, University Hospital of Guadeloupe, Pointe-à-Pitre, Guadeloupe France

## Abstract

**Objectives:**

The incidence of head and neck squamous cell carcinoma (HNSCC) in the French West Indies (FWI) is relatively high, despite a low prevalence of tobacco smoking and alcohol drinking. Little is known about other risk factors in the FWI. We assessed associations between several factors and HNSCC risk, their population attributable fractions (PAF) in the FWI, and compared these PAFs by subsite, sex and age.

**Materials and methods:**

We conducted a population-based case-control study (145 cases and 405 controls). We used logistic regression models to estimate adjusted odds-ratios (OR), PAFs and their 95% confidence intervals (CI).

**Results:**

Tobacco smoking, alcohol drinking, high-risk HPV, family history of HNC, low BMI and several occupations and industries were significantly associated to the occurrence of HNSCC. The majority of HNSCC cases were attributable to tobacco smoking (65.7%) and alcohol (44.3%). The PAF for the combined consumption of tobacco and/or alcohol was 78.2% and was considerably larger in men (85%) than in women (33%). The PAFs for the remaining risk factors were 9% for family history of HNSCC, 9% for low BMI, 15% for high-risk HPV, and 25% for occupations. The overall PAF for all risk factors combined was 89.0% (95% CI = 82.0–93.2). The combined PAFs by sex were significantly greater in men (93.4%, 95% CI = 87.5–96.5) than in women (56.4%, 95% CI = 18.7–76.6).

**Conclusion:**

Tobacco and alcohol appeared to have the greatest impact on HNSCC incidence among the studied risk factors, especially among men. Prevention programs for HNSCC in the FWI should target tobacco and alcohol cessation, particularly in men. Future research should emphasise on the role of occupational factors to better understand this disease.

**Supplementary Information:**

The online version contains supplementary material available at 10.1186/s12885-021-08787-4.

## Introduction

Head and neck cancer is a public health concern across the world, counting 700,000 new cases every year [1]. Tobacco smoking and alcohol drinking are the major risk factors. However, in Guadeloupe and Martinique, two French overseas territories in the French West Indies (FWI), the prevalence of these risk factors is relatively low (27 and 6% respectively) [2] whereas incidence rates of HNC among men are among the highest in Latin America and the Caribbean [1]. In 2018, age-standardized (world) incidence rates of head and neck cancer per 100,000 were estimated to be 8.1 in Guadeloupe (men: 15.5; women: 2.1) and 5.7 in Martinique (men: 12.1; women: 0.6) [1]. Thus, other risk factors known or suspected to be associated with an increased risk of HNC may be contributing actively to the cancer burden in these regions. Risk factors that have been previously found to be associated with an increased risk of head and neck cancer are infection with human papillomavirus (HPV), low vegetable and fruit consumption, low body mass index (BMI), occupational exposures and family history of HNC [3–14]. We previously demonstrated that in this population high-risk oral human papillomavirus (Hr-HPV) infections were associated with an increase in head and neck squamous cell carcinoma risk (HNSCC) [15, 16].

Estimating population attributable fractions (PAF) of the different risk factors of HNSCC could be used to attain a better understanding of the public health impact provided that these risk factors are established causes of HNSCC [17]. Furthermore, these PAFs are subject to geographic variation considering the relationship between the frequency of the studied risk factor and the socio-cultural context; therefore, warranting population-specific assessments.

Previous studies have looked at the PAFs for tobacco and alcohol [18–20]. In particular, they were responsible for 72% of these cancers in an international pooled analysis [20]. The effects of tobacco and alcohol on HNSCC risk have also been reported in the FWI and other black populations but the PAFs were not assessed [16, 21, 22]. In addition, some studies have attempted to address the proportion of cases attributable to other risk factors [18, 23, 24]. However, our knowledge on other known or suspected risk factors and their public health impact is still limited and particularly scarce in populations of African descent [25]. While studying PAFs for suspected risk factors that are not recognized causes of the disease presents issues in interpretation, these estimates may be beneficial for setting priorities for cancer prevention. Impact measures for these factors are notably crucial in the FWI where the prevalence of the main causal risk factors are lower compared to mainland France [2] and other European countries where HNSCC incidence is also high.

This is the first study to investigate the impact of known or suspected risk factors of HNSCC in an Afro-Caribbean population. We aimed to investigate the role and impact of several HNSCC risk factors (tobacco, alcohol, family history of HNC, diet, low BMI, Hr-HPV and at-risk occupations) in the French West Indian population. We assessed the associations between these risk factors and HNSCC risk. In addition, we estimated population attributable fractions and we compared these PAFs in different subgroups of the study population. This knowledge could have substantial implications for the prevention of head and neck cancers in the FWI. In particular, identify specific situations for the implementation of primary prevention or screening programs, and provide useful data to assess the potential impact of HPV vaccination.

## Methods

### Study population, data and specimen collection

We conducted a population-based case-control study in Martinique and Guadeloupe. The study is an extension of a large nationwide case-control study, the ICARE study, which has already been conducted in ten French regions covered by a cancer registry [26]. The study in the FWI used the same protocol and questionnaire, described in detail elsewhere [26], with some adaptations to the local context. Eligible cases were patients residing in the FWI, suffering from a primary, malignant tumour of the oral cavity, pharynx, sinonasal cavities and larynx of any histological type, aged between 18 and 75 years old at diagnosis, newly diagnosed and histologically confirmed between April 1, 2013 and June 30, 2016. Incident cases were identified in collaboration with the population-based cancer registries of Guadeloupe and Martinique which use standardized procedures for the recording of all cancer cases in each of these regions. Our study benefitted from diverse data sources of the registries in order to flag new cases diagnosed during the study period. The control group was selected from the general population by random digit dialling, using the incidence density sampling method. Controls were frequency matched to the cases by sex, age and region. Additional stratification was used to achieve a distribution by socioeconomic status among the controls comparable to that of the general population.

Cases and controls were interviewed face-to-face. The questionnaire included information on sociodemographic characteristics, lifetime tobacco smoking, lifetime alcohol consumption, history of cancer among first-degree relatives, usual diet, anthropometric variables (height, weight at different points in time), hormonal factors, and lifetime occupational history. Participants were also asked to provide a saliva sample, using the Oragene® OG-500 kit (DNA Genotek).

Among the 235 eligible cases, 170 (72.3%) agreed to participate and were interviewed. Among the 497 eligible controls, 405 (81.5%) answered the questionnaire. Among cases and controls with interview data, 114 cases (67.1%) and 311 controls (76.8%) provided a saliva sample. Written informed consent was provided by all participants in the study and all data recorded was anonymised prior to analysis. Ethics approval was granted to this study by the French Data Protection Authority (CNIL, Commission Nationale de l’Informatique et des Libertés) n° DR-2015-2027; and the institutional review board of the French National Institute of Health and Medical Research (IRB INSERM n°01–036).

### HPV detection and genotyping

The detection of HPV-integrated DNA from saliva samples was performed with the INNO-LiPA® kit, according to the manufacturer’s instructions (INNO-LiPA HPV Genotyping *Extra*; Innogenetics, Ghent, Belgium). The INNO-LiPA HPV genotyping assay allows the detection of the following genotypes: HPV16, HPV18, HPV31, HPV33, HPV35, HPV39, HPV45, HPV51, HPV52, HPV56, HPV58, HPV59, HPV68 (High-risk), HPV26, HPV53, HPV66, HPV70, HPV73, HPV82 (Probable high-risk), HPV06, HPV11, HPV40, HPV42, HPV43, HPV44, HPV54, HPV61, HPV81 (Low-risk), HPV62, HPV67, HPV83, HPV89 (Other). The full details on the method for HPV detection has been described elsewhere [15].

### Exposure variables

The definitions for our variables were adopted from previous work derived from this case-control study and the ICARE study where applicable [15, 26]. Ever cigarette smokers were defined as persons who smoked at least 100 cigarettes in their lifetime. Ever daily alcohol drinking was defined as at least one glass per day during at least 1 year.

To ascertain the family history of HNC cancer, subjects were first asked to indicate whether any of their first-degree relatives (biological mother and father, and full brothers or sisters) were diagnosed with head and neck cancer. No verification of the cancer diagnosis in the relatives was performed.

We examined the relationship between BMI at different time points (at interview, 2 years before the interview and at age 30). BMI was computed as weight (kg) divided by height squared (m^2^). In relation to BMI, the study population was divided into four categories according to the World Health Organization (WHO) international classification [27]: underweight subjects (BMI < 18.5 kg/m^2^), subjects with normal weight (18.5 kg/m^2^ ≥ BMI < 24.9 kg/m^2^), overweight subjects (25.0 kg/m^2^ ≥ BMI < 29.9 kg/m^2^), and obese subjects (BMI ≥ 30 kg/m^2^).

Oral Hr-HPV status was assessed as high-risk-HPV-positive versus high-risk-HPV-negative, the latter category grouping HPV-negative and non-high-risk-HPV genotypes. Participants were as defined Hr-HPV-positive when at least one high-risk HPV type was detected in the saliva sample that they provided.

Occupational exposures were ascertained by collecting detailed lifetime job history during the interview. The international Standard Classification of Occupations (ISCO) and the French Nomenclature of Activities (NAF) were used by a trained coder to blindly code occupations and branches of the industry, independently of the case-control status of the participants [28, 29].

Information on diet was collected using a food frequency questionnaire [30]. We developed beforehand a list of food items pertinent to our research question and/or consumed regularly in the French West Indies. Participants were asked whether or not they consumed one of the foods in the list, then they were asked to specify the usual frequency at which they consumed those foods.

Information on menstruation, menopause, reproductive characteristics (pregnancies, live births, miscarriages and abortions), lifelong use of oral contraceptives, and hormone replacement therapy (HRT) were recorded exclusively for female participants. HRT was defined as hormone therapy intended to treat menopausal symptoms.

### Statistical analysis

The current analysis was restricted to squamous cell carcinomas of the oral cavity (International Classification of Diseases 10th revision codes C00.3-C00.9, C02.0-C02.3, C03.0, C03.1, C03.9, C04.1, C04.8, C04.9, C05.0, C06.0-C06.2, C06.8 and C06.9, *n* = 35), the oropharynx (ICD-10 codes C01.9, C02.4, C05.1, C05.2, C09, C10, C 14.2, *n* = 58), the hypopharynx (ICD-10 codes C12- C13, *n* = 19) and the larynx (ICD-10 codes C32, *n* = 32). Our analysis included 145 cases and 405 controls. The association between the various risk factors (ever tobacco, ever daily alcohol, HPV, low vegetable and fruit consumption, low body mass index, occupational exposures and family history of HNC) and the occurrence of HNSCC was assessed by estimating odds ratios (ORs) adjusted for age, sex and recruitment site, and their 95% confidence intervals (CIs), using logistic regression models. Further adjustment for educational level (primary or less, vocational secondary, general secondary and university) did not modify any of the estimates and was not included in the models. An analysis on the job history of subjects were conducted beforehand to assess the association between the occurrence of HNSCC and having held a certain occupation at least once in the participant’s lifetime regardless of the duration. We then created a single variable to take into account the overall risk associated with occupation. Someone who had an at-risk occupational activity was defined as someone who held a job at least once during his/her lifetime in one or more of the occupations which were significantly associated with HNSCC risk based on our analyses, and evidence in the literature. The occupations and sectors selected to construct this variable were: cook (ISCO 53130), banana plantation worker (ISCO 62210 and NAF 01.1F), mason, carpenter, and other construction workers (ISCO 95), labourers (ISCO 99) and workers in the manufacture of metal products (NAF 28). The ORs for the associations between HNSCC and the occupations and sector used to construct the at-risk occupational activity variable are available in the supplementary Table 1. The at-risk occupational activity variable was used in the final model to calculate PAFs.

The association between HNSCC and each risk factor was assessed prior to the final logistic regression model to calculate the PAFs. Firstly, each risk factor was regressed individually adjusting for age, sex and region. Tobacco was further adjusted for alcohol and vice-versa. The multiplicative interaction between ever tobacco smokers and daily drinkers was non-significant, and thus, the cross-product term was not included in the model. All remaining factors were further adjusted for tobacco and alcohol. Secondly, another logistic regression model was then fit with all the significant risk factors as binary variables simultaneously. For each risk factor, the PAF was calculated from the adjusted ORs and the proportion of exposed among cases (PE_c_) as PAF = PE_c_ x (OR-1)/OR. The PAFs as well as their 95% CI were computed using the aflogit procedure [31] available in STATA software (StataCorp, Texas, USA). This procedure is based on the method of Greenland and Drescher for the logistic model adapted to case–control studies [31].

The PAFs were also calculated in different subgroups: in men and in women, in oropharyngeal and non-oropharyngeal cancer, and in persons < 59 years and ≥ 59 years. One case had multiple simultaneous tumours located in the hypopharynx and oropharynx, and was excluded from the analyses by subsite.

Missing data were observed for HPV status (53 cases, 97 controls), family history of HNC (24 cases, 4 controls), BMI (23 cases, 4 controls) and smoking status (one case). We used multiple imputations by chained equations to deal with missing data [32]. The imputation model contained all the basic characteristics of the participants (age, sex recruitment site and education level), alcohol and smoking related variables (ever daily alcohol drinking, quantity of alcohol, smoking status, smoking duration, and smoking quantity), HPV status (low-risk, probable high-risk, high-risk, and other HPV types), BMI, family history of cancer and the case-control status. All variables in the imputation model which had missing values were imputed for our analyses. We generated 20 datasets. We combined the estimates and their variances/covariances into one data set using the pooling algorithm suggested by Rubin et al. to perform statistical inferences [33]. We also performed a complete case analysis, on the dataset containing only observed data (Supplementary Table S2 and Table S3).

## Results

We found no significant association between HNSCC risk and the consumption of fruits and/or vegetables. The highest OR was found among those who consumed fruits and vegetables less than once a week (OR = 1.46 95%CI = 0.61–3.51), compared to a consumption of at least once a week. These variables did not reach statistical significance and were not included in the final model.

Table 1 shows ORs and PAFs for HNSCC associated with the other risk factors. Tobacco smoking and daily alcohol drinking, family history, low BMI and certain occupations and industries increased significantly the risk of HNSCC. Overall, ever tobacco smoking and daily alcohol drinking were associated with the occurrence of HNSCC (OR = 5.86 95%CI = 3.47–9.90 and OR = 3.03 95%CI = 1.85–4.95 respectively). The PAFs were 65.8% for tobacco smoking, 43.3% for alcohol drinking. The PAF for the combined consumption of alcohol and/or tobacco, was 78.1% (95% CI = 68.3–85.0).

**Table 1:** Adjusted odds ratios (OR), population attributable fractions (PAF) and 95% confidence intervals (CI) for HNSCC associated with tobacco, alcohol, family history of HNC, low BMI, Hr-HPV and at-risk occupations

Risk factor	Cases	Controls						
	*n*	*n*	OR^a^	95% CI^a^	OR^b^	95% CI^b^	PAF^b^	95% CI^b^
Tobacco smoking								
Ever	114	142	6.72	(4.24–10.64)	5.86	(3.47–9.90)	65.7%	(51.7–75.6)
Never	30	263	1	ref	1	ref	1	ref
Missing	1	0						
Daily alcohol								
Ever	96	112	4.95	(3.24–7.58)	3.03	(1.85–4.95)	44.3%	(27.3–57.3)
Never	49	293	1	ref	1	ref	1	ref
Missing	0	0						
Family history of HNC								
Yes	13	14	3.67	(1.66–8.08)	4.70	(1.81–12.22)	8.9%	(1.1–16.1)
No	108	387	1	ref	1	ref	1	ref
Missing	24	4						
Body mass index								
BMI < 18.5	12	10	4.63	(1.89–11.33)	4.96	(1.69–14.59)	9.0%	(1.2–16.1)
BMI ≥ 18.5	110	391	1	ref	1	ref	1	ref
Missing	23	4						
Oral HPV Status								
Hr-HPV+	19	30	2.31	(1.34–3.99)	2.39	(1.23–4.63)	15.1%	(3.9–25.0)
Hr-HPV-	73	277	1	ref	1	ref	1	ref
Missing	53	98						
At-risk occupational activity								
Ever	56	87	2.08	(1.37–3.17)	2.78	(1.66–4.66)	24.7%	(12.1–35.5)
Never	89	318	1	ref	1	ref	1	ref
Missing	0	0						

French West Indies, 2013–2016

Analyses performed using imputed data (145 cases 405 controls)

^a^Adjusted for age, sex, and region (variables used for frequency matching)

^b^Adjusted for age, sex, region and all the risk factors in the table (ever tobacco, ever daily alcohol, Hr-HPV, low body mass index, family history of HNC and at-risk occupational activity)

Family history of HNC was associated with a four-fold increase in HNSCC risk and 8.9% of the cases overall were attributable to this risk factor. Overall, 9.0% of cases were attributable to low BMI. Overall, 15.1% of cases were attributable to Hr-HPV. The PAF for Hr-HPV was similar among oropharyngeal cases (12.1%, 95% CI = -5.4–26.6) and non-oropharyngeal cases (14.3%, 95% CI = -0.9–27.2). Overall, 24.7% of cases were attributable to at-risk occupational activity.

The PAF for all risk factors combined (Table 2) was 89.0% (95% CI = 82.0–93.2). On one hand, the combined PAF was different by sex and by age group. PAF were significantly greater in men (93.4%, 95% CI = 87.5–96.5) than in women (56.4%, 95% CI = 18.1–76.6) and were slightly more elevated in younger persons (< 59 y, 94.2%) than in older persons (≥59 y, 84.9%). On the other hand, no difference was found by subsite. The PAF for was 88.5% in oropharyngeal cancers and 90.0% in non-oropharyngeal cancer. Differences by subgroup were mainly due to differences in the PAF for tobacco, and also for alcohol exclusively between men and women. The PAF for the combined consumption of tobacco and/or alcohol was considerably larger in men (PAF = 84.8%, 95% CI = 75.7–90.5) than in women (PAF = 32.9%, 95% CI = -13.8− 60.4).

**Table 2:** Population attributable fractions (PAF) and 95% confidence intervals (CI) of all risk factors combined for HNSCC, overall and by subgroups

	Cases	Controls		
	*n*	*n*	PAF	95% CI
HNSCC	145	405	89.0%	(82.0–93.2)
By subsite				
Oropharynx	58	405	88.5%	(75.9–94.5)
Non-oropharynx	86	405	90.0%	(81.2–94.7)
By Sex				
Men	127	306	93.4%	(87.5–96.5)
Women	18	99	56.4%	(18.1–76.6)
By age				
< 59	67	203	94.2%	(85.5–97.7)
≥ 59	78	202	84.9%	(72.5–91.7)

French West Indies, 2013–2016

Analyses performed using imputed data (145 cases 405 controls)

ORs estimates and PAFs for tobacco, alcohol, family history, BMI, Hr-HPV status and occupation were widely similar between analyses generated from our observed data and our imputed data. Point estimates for ORs from the observed data set were slightly higher for alcohol (OR = 4.29, 95%CI = 2.28–8.07) and BMI (OR = 6.96, 95%CI = 1.98–24.52), slightly lower for tobacco (OR = 4.94, 95%CI = 2.52–9.66). (Supplementary Table S2 and Table S3).

We performed an exploratory analysis on the role of hormonal factors in the occurrence of HNSCC on the subgroup of 117 women (18 cases and 99 controls). Although the measures of association were not systematically significant, exogenous and endogenous exposure to hormonal factors were found to be consistently associated with a decreased risk of HNSCC in women. Compared to women who used oral contraceptives, never users were found to be at a greater risk for HNSCC. Shorter lifetime menstruation (begin after 13 and end ≤50 years old) was observed to be significantly associated with an increase in HNSCC risk compared to longer periods of lifetime menstruation (OR = 22.35, 95% CI = 3.28–152.28). In terms of reproductive factors, giving birth to no children was associated with a non-significant increase in risk of HNSCC compared to those who had 2 or more (OR = 8.08, 95%CI = 0.92–71.34). Although non-significant, women who never miscarried a child were also at a greater risk for HNSCC (OR = 2.97, 95%CI = 0.62–14.12) (Supplementary Table S5).

## Discussion

This is the first study to investigate the impact of known or suspected risk factors of HNSCC in an Afro-Caribbean population. We were able to attribute 89% of the HNSCC cases to the studied risk factors in this paper, and highlighted the predominant impact of tobacco smoking and alcohol drinking on HNSCC incidence, especially among men.

The combined consumption of tobacco and alcohol drinking accounted for the largest proportion of cases (78%). Despite a low prevalence of tobacco smoking and alcohol drinking in the FWI [34] our results for predominant impacts of tobacco and alcohol were consistent with other studies [18–20, 35, 36].

Family history of HNC among first-degree relatives, low BMI, and some occupations and industries were also significantly associated to HNSCC risk. These findings coincide with several other studies [4, 10–12, 23, 37–40].

The increased HNSCC risk among banana plantations workers is a novel finding, as this occupation can only be investigated in a limited number of populations, and requires further analysis, given the extensive use of chlordecone and other pesticides in banana farming in the FWI [41–43]. Contrarily to previous studies [18, 44–47], our current work did not reveal any significant associations with consumption of fruits and/or vegetables, after adjusting for other risk factors.

Regarding the proportion of preventable cases for these significant risk factors, the PAFs ranged from 9 to 25%. Previous studies investigated mainly PAF for tobacco and alcohol; however, those who looked at other risk factors reported PAFs which were of similar order of magnitude to ours [18, 23]. Occupational exposure accounted for 25% of the cases in our sample, and this PAF was greater than what was estimated previously in an international study [38]. The proportion of cases attributable to family history of HNC in the FWI was higher than what was reported by a pooled analysis from the INHANCE consortium and two European studies [18, 23, 37].

The PAF for oral Hr-HPV was overall 15%. Other studies reported global attributable fractions for Hr-HPV which were consistently greater for the oropharynx (between 21.3 and 30.8%) than the other the other subsites separately [24, 48, 49]. Our results, on the hand, showed no major differences by subsites (12% for oropharynx and 14% for oral cavity, hypopharynx and larynx together) and the PAFs for Hr-HPV in the oropharynx was lower than in other studies. Although the aetiological fraction of Hr-HPV in the FWI was not as substantial as that of tobacco and alcohol, a noteworthy proportion of cases could be attributed to Hr-HPV infections and therefore, this population could still draw considerable benefits from primary cancer prevention through HPV vaccination [50].

Similarly to previous studies, great sex disparities were observed in the proportion of cases attributable by all the factors studied initially (93.4% in men and 56.4% in women) [18, 19]. This difference is due to the low prevalence of tobacco and alcohol in women, as well as weaker associations. Our results on hormonal factors in women coincide with previous studies which show that exposure to oestrogen reduces the risk of HNSCC [51–53].

We acknowledge that PAFs are conventionally calculated for risk factors with an established causal link with the disease. In addition, some of the factors studied are indeed non-modifiable and may not provide many avenues for prevention and control, especially in regards to BMI, and the underlying health concerns which may arise from recommending weight gain in the population. However, looking at known or suspected risk factors could contribute towards a better understanding of the aetiology of HNSCC in the FWI population and assist in decision-making for public health interventions.

Our study presents limitations. We had a small sample size and limited the possibilities of analysis by subgroup. The risk factors were ascertained mostly by using self-reported measures and may have induced misclassification bias. We cannot disregard the possibility of a recall bias due to the retrospective study design. It was shown however that participants in case-control studies tend to report accurately information on cancer in first-degree relatives [54, 55]. Furthermore, BMI from 2 years prior to the interview was used to avoid underestimating the BMI due to weight loss associated with head and neck cancer diagnosis. In our study we were able to investigate a large number of known or suspected risk factors of HNSCC that were studied in previous reports [18–20, 35, 36]. Consequently, we were able to explore various areas such as hormonal factors in women. Occupational exposures were assessed collectively as one variable based on occupation and thus, we are unable to produce any information for aetiological fractions for specific occupational exposures.

We had 27% missing data for HPV in our sample. To handle missing data, we used a multiple imputation procedure shown to produce less biased and more precise estimates rather than excluding individuals with missing data [32].. Indeed the estimates generated by this method were similar to those from the observed data set (supplementary Table S2 and Table S3). Furthermore, the use of oral HPV detection to assess the HPV status may have resulted in misclassification, which is however likely to be non-differential. Oral HPV detection has been shown to have good specificity but moderate sensitivity for HPV-positive HNSCC tumours [56].

We were able to produce a representative sample of cases and controls. We compared the distribution for sex, age and cancer sites of our cases with data from the population-based cancer registries of Guadeloupe and Martinique, and observed a concordance with HNC cases in the French West Indies. The control group was constituted using a method from a similar study which yielded unbiased samples; thus our controls could be considered representative of the general population of similar age and sex [26]. We also confirmed the representativeness of the tobacco, alcohol and BMI distribution in our control group to FWI population after comparison with the data from a national health survey [34]. Eleven percent of cases could be attributable to residual risk factors that were not taken into account during our study. Factors like gene-environment interactions and medical history were not studied and could bring further clarification to HNSCC aetiology in the FWI.

## Conclusion

This is first time the impact of tobacco, alcohol, oral HPV, low BMI, family history of HNC and at-risk occupational activity have been studied in an Afro-Caribbean population. Overall, we were able to explain 89.0% of HNSCC in the FWI based on the risk factors studied in this report. Tobacco and alcohol appeared to have the greatest impact on HNSCC incidence among the other risk factors (78.1%). Given the large attributable fraction for occupational risk factors (24.7%) the public health impact could be considerable if we reduced these exposures. Special attention should be given to tobacco and alcohol cessation in particular in men and younger persons, when considering prevention programs for HNSCC in the FWI. More in-depth analyses are warranted on occupational exposures in the FWI, and future research on HNSCC should emphasise on the role of hormonal factors to better understand this disease in women.

## Additional file

**Additional file 1: Table S1.** Adjusted ORs for the individual occupations and sectors used to construct the occupational exposure variable. **Table S2.** Adjusted odds ratios (OR), population attributable fractions (PAF) and 95% confidence intervals (CI) for HNSCC associated with tobacco, alcohol, family history of HNC, low BMI, Hr-HPV and at-risk occupations (observed data). **Table S3.** Population attributable fractions (PAF) and 95% confidence intervals (CI) of all risk factors combined for HNSCC, overall and by subgroups (observed data). **Table S4.** Adjusted odds ratios (OR) and 95% confidence intervals (CI) for HNSCC associated with hormonal factors among women (observed data). **Table S5.** Adjusted odds ratios (OR) and 95% confidence intervals (CI) for HNSCC associated with hormonal factors among women (**Imputed data**).

## Data Availability

The data that support the findings of this study are available from the corresponding author upon reasonable request.

## Abbreviations

BMI: Body mass index
CI: Confidence interval
FWI: French West Indies
HNC: Head and neck cancer
HNSCC: Head and neck squamous cell carcinoma
HPV: Human papillomavirus
Hr-HPV: High-risk human papillomavirus
IARC: International Agency for Research on Cancer
INHANCE: International Head and Neck Cancer Epidemiology Consortium
MICE: Multiple imputation by chained equations
OR: Odds ratio
PAF: Population attributable fraction
