# Supplementary material for: Head and neck cancer risk factors in the French West Indies
Source: BMC Cancer. 2021 Sep 30;21:1071. doi: 10.1186/s12885-021-08787-4 (PMC8482677; doi:10.1186/s12885-021-08787-4)
Supplement: Supplementary file 1 — Additional file 1: Table S1. Adjusted ORs for the individual occupations and sectors used to construct the occupational exposure variable. Table S2. Adjusted odds ratios (OR), population attributable fractions (PAF) and 95% confidence intervals (CI) for HNSCC associated with tobacco, alcohol, family history of HNC, low BMI, Hr-HPV and at-risk occupations (observed data). Table S3. Population attributable fractions (PAF) and 95% confidence intervals (CI) of all risk factors combined for HNSCC, overall and by subgroups (observed data). Table S4. Adjusted odds ratios (OR) and 95% confidence intervals (CI) for HNSCC associated with hormonal factors among women (observed data). Table S5. Adjusted odds ratios (OR) and 95% confidence intervals (CI) for HNSCC associated with hormonal factors among women (Imputed data). [file 12885_2021_8787_MOESM1_ESM.pdf]

Title: Head and neck cancer risk factors in the French West Indies.

Author list:

- Aviane Auguste
- Clarisse Joachim
- Jacqueline Deloumeaux
- Stanie Gaete
- Leah Michineau
- Cécile Herrmann-Storck
- Suzy Duflo
- Danièle Luce

Supporting information

Multivariate logistic regression analyses performed on occupational exposures, sensitivity analysis and hormonal factors in women

S1 table: Adjusted ORs for the individual occupations and sectors used to construct the occupational exposure variable.

ISCO code	NAF code	Title	Cases *n*	Controls *n*	OR†	95% CI
		**At-risk occupational activity**	56	87	2.74	(1.61-4.67)
ISCO		***Occupation***				
53130	Any	Cook, except private service	7	8	5.22	(1.64-16.67)
62210	01.1F	Banana plantation workers	9	6	3.89	(0.96-15.74)
95	Any	Bricklayers, Carpenters and Other Construction Workers	35	58	1.87	(1.02-3.44)
99	Any	Labourer	14	22	2.34	(0.98-5.61)
	NAF	***Sector***				
Any	28	Manufacture of fabricated metal products, except machinery and equipment	7	6	6.52	(1.69-25.14)

†: Adjusted for age, sex, region, the combination of cigarette smoking duration and intensity (cigarette/day), and daily alcohol drinking

S2 table: Adjusted odds ratios (OR), population attributable fractions (PAF) and 95% confidence intervals (CI) for HNSCC associated with tobacco, alcohol, family history of HNC, low BMI, Hr-HPV and at-risk occupations (observed data).

Risk factor	Cases *n*	Controls *n*	OR†	95% CI	PAF	95% CI
**Tobacco smoking**						
Ever	65	107	4.94	(2.52-9.66)	62.5%	(41.3-76.0)
Never	18	197				
**Daily alcohol**						
Ever	60	84	4.29	(2.28-8.07)	55.4%	(34.7-69.6)
Never	23	220				
**Family history of HNC cancer**						
Yes	8	10	4.29	(1.30-14.17)	7.4%	(-3.2-16.9)
No	75	294				
**Body mass index**						
BMI< 18.5	11	7	6.96	(1.98-24.52)	11.4%	(0.7-20.8)
BMI≥ 18.5	72	297				
**Oral HPV Status**						
Hr-HPV+	19	30	2.49	(1.16-5.35)	13.7%	(-0.5-26.0)
Hr-HPV-	64	274				
**At-risk occupational activity**						
Ever	34	68	2.94	(1.52-5.68)	27.0%	(9.7-41.0)
Never	49	236				

French West Indies, 2013-2016

Analysis performed on 387 subjects (cases=83, controls=304). Subjects with missing data were removed from the analysis.

†: Adjusted for age, sex, region and all the risk factors in the table (ever tobacco, ever daily alcohol, Hr-HPV, low body mass index, family history of HNC and at-risk occupational activity)

S3 table: Population attributable fractions (PAF) and 95% confidence intervals (CI) of all risk factors combined for HNSCC, overall and by subgroups (observed data).

	Cases *n*	Controls *n*	PAF	95% CI
HNSCC	83	304	90.1%	(81.1-94.8)
By subsite				
Oropharynx	37	304	90.2%	(74.7-96.2)
Non-oropharynx	45	304	90.9%	(78.2-96.2)
By Sex				
Men	72	225	93.9%	(85.8-97.4)
Women	11	79	64.6%	(13.1-85.6)
By age				
<59	40	152	93.9%	(81.3-98.0)
≥59	43	152	86.8%	(69.5-94.3)

French West Indies, 2013-2016.
Analysis performed on 387 subjects (cases=83, controls=304). Subjects with missing data were removed from the analysis.

S4 table: Adjusted odds ratios (OR) and 95% confidence intervals (CI) for HNSCC associated with hormonal factors among women (observed data).

Hormonal factor	Case		Control			
	n	col%	*n*	col%	OR†	95% CI
Exogeneous factors						
**Oral contraceptive use**						
Ever	9	(64.3)	83	(84.7)	1	ref
Never	5	(35.7)	15	(15.3)	2.27	(0.60-8.64)
Missing	4		1			
**Hormone replacement therapy (HRT)**						
Ever	1	(7.7)	23	(35.9)	1	ref
Never	12	(92.3)	41	(64.1)	5.39	(0.61-47.54)
Missing	5		35			
Endogeneous factors						
**Age at Menarche**						
≤ 13	5	(35.7)	67	(68.4)	1	ref
over 13	9	(64.3)	31	(31.6)	4.95	(1.30-18.92)
Missing	4		1			
**Period of menstruation (binary)** ‡						
Begin after 13 and end ≤ 50 years old	8	(61.5)	12	(20.3)	26.49	(3.69-189.93)
Other scenarios	5	(37.8)	47	(79.7)	1	ref
Missing	5		40			
**Ever Pregnant**						
Yes	9	(75.0)	91	(93.8)	1	ref
No	3	(25.0)	6	(6.2)	5.92	(0.88-39.88)
Missing	6		2			
**Parity**						
No children	3	(23.1)	6	(6.4)	8.23	(0.96-70.50)
1 child	3	(23.1)	15	(16.0)	8.75	(1.13-67.71)
2 children	3	(23.1)	28	(29.8)	1.53	(0.26-8.99)
> 2 children	4	(30.8)	45	(47.9)	1	ref
Missing	5		5			
**Miscarriage**						
Ever	2	(16.7)	38	(41.3)	1	ref
Never	10	(83.3)	54	(58.7)	3.94	(0.68-22.84)
Missing	6		7			

French West Indies, 2013-2016.

Analysis performed on observed data. Subjects with missing data were removed from the analysis.

†: Adjusted for age, sex, region, smoking status (current/former), daily alcohol drinking

‡: Age at first menarche and menopause - “shorter lifetime menstruation”: Begin after 13 – end ≤ 50. “Other scenarios”: aggregation of the following categories for ≤ 13 – end ≤ 50; after 13 – end after 50; ≤ 13 – end after 50.

S5 table: Adjusted odds ratios (OR) and 95% confidence intervals (CI) for HNSCC associated with hormonal factors among women (**Imputed data**).

**Hormonal factor**	**Case**		**Control**			
	n	**col%**	***n***	**col%**	**OR**†	**95% CI**
Exogeneous factors						
**Oral contraceptive use**						
Ever	9	(64.3)	83	(84.7)	1	ref
Never	5	(35.7)	15	(15.3)	2.68	(0.71-10.18)
Missing	4		1			
**Hormone replacement therapy (HRT)**						
Ever	1	(7.7)	23	(35.9)	1	ref
Never	12	(92.3)	41	(64.1)	3.66	(0.55-24.56)
Missing	5		35			
Endogeneous factors						
**Age at Menarche**						
≤ 13	5	(35.7)	67	(68.4)	1	ref
over 13	9	(64.3)	31	(31.6)	4.79	(1.26-18.27)
Missing	4		1			
**Period of menstruation (binary)** ‡						
Begin after 13 and end ≤ 50 years old	8	(61.5)	12	(20.3)	22.35	(3.28-152.28)
Other scenarios	5	(37.8)	47	(79.7)	1	ref
Missing	5		40			
**Ever Pregnant**						
Yes	9	(75.0)	91	(93.8)	1	ref
No	3	(25.0)	6	(6.2)	2.48	(0.49-12.54)
Missing	6		2			
**Parity**						
No children	3	(23.1)	6	(6.4)	8.08	(0.92-71.34)
1 child	3	(23.1)	15	(16.0)	6.28	(0.87-45.61)
2 children	3	(23.1)	28	(29.8)	1.59	(0.29-8.73)
> 2 children	4	(30.8)	45	(47.9)	1	ref
Missing	5		5			
**Miscarriage**						
Ever	2	(16.7)	38	(41.3)	1	ref
Never	10	(83.3)	54	(58.7)	2.97	(0.62-14.12)
Missing	6		7			

French West Indies, 2013-2016.
Analysis performed on 117 women (18 cases and 99 controls).
†: Adjusted for age, sex, region, smoking status (current/former), daily alcohol drinking
‡: Age at first menarche and menopause - "shorter lifetime menstruation": Begin after 13 – end ≤ 50. "Other scenarios": aggregation of the following categories for ≤ 13 – end ≤ 50; after 13 – end after 50; ≤ 13 – end after 50.
